# Using Latent Class Analyses to Examine Health Disparities among Young Children in Socially Disadvantaged Families during the COVID-19 Pandemic

**DOI:** 10.3390/ijerph19137893

**Published:** 2022-06-27

**Authors:** Rosa S. Wong, Keith T. S. Tung, Nirmala Rao, Ko Ling Chan, King-Wa Fu, Jason C. Yam, Winnie W. Y. Tso, Wilfred H. S. Wong, Terry Y. S. Lum, Ian C. K. Wong, Patrick Ip

**Affiliations:** 1 Department of Paediatrics and Adolescent Medicine, The University of Hong Kong, Hong Kong SAR, China; rosawg@connect.hku.hk (R.S.W.); keith-tung@connect.hku.hk (K.T.S.T.); wytso@hku.hk (W.W.Y.T.); whswong@hku.hk (W.H.S.W.); 2 Department of Social Work and Social Administration, The University of Hong Kong, Hong Kong SAR, China; tlum@hku.hk; 3 Department of Pharmacology and Pharmacy, The University of Hong Kong, Hong Kong SAR, China; wongick@hku.hk; 4 Faculty of Education, The University of Hong Kong, Hong Kong SAR, China; nrao@hku.hk; 5 Department of Applied Social Sciences, The Hong Kong Polytechnic University, Hong Kong SAR, China; koling.chan@polyu.edu.hk; 6 Journalism and Media Studies Centre, The University of Hong Kong, Pokfulam, Hong Kong SAR, China; kwfu@hku.hk; 7 Department of Ophthalmology and Visual Sciences, The Chinese University of Hong Kong, Hong Kong SAR, China; yamcheuksing@cuhk.edu.hk; 8 State Key Laboratory of Brain and Cognitive Sciences, The University of Hong Kong, Hong Kong SAR, China; 9 Research Department of Practice and Policy, UCL School of Pharmacy, London WC1N 1AX, UK

**Keywords:** COVID-19, preschooler, health disparity, latent class analysis, family hardship

## Abstract

Rising income inequality is strongly linked to health disparities, particularly in regions where uneven distribution of wealth and income has long been a concern. Despite emerging evidence of COVID-19-related health inequalities for adults, limited evidence is available for children and their parents. This study aimed to explore subtypes of families of preschoolers living in the disadvantaged neighborhoods of Hong Kong based on patterns of family hardship and to compare their patterns of parenting behavior, lifestyle practices, and wellbeing during the COVID-19 pandemic. Data were collected from 1338 preschoolers and their parents during March to June 2020. Latent class analysis was performed based on 11 socioeconomic and disease indicators. Multivariate logistic regressions were used to examine associations between identified classes and variables of interest during the COVID-19 pandemic. Four classes of family hardship were identified. Class 1 (45.7%) had the lowest disease and financial burden. Class 2 (14.0%) had the highest financial burden. Class 3 (5.9%) had the highest disease burden. Class 4 (34.5%) had low family income but did not receive government welfare assistance. Class 1 (low hardship) had lower risks of child maltreatment and adjustment problems than Class 2 (poverty) and Class 3 (poor health). However, children in Class 1 (low hardship) had higher odds of suffering psychological aggression and poorer physical wellbeing than those in Class 4 (low income), even after adjusting for child age and gender. The findings emphasize the need to adopt flexible intervention strategies in the time of large disease outbreak to address diverse problems and concerns among socially disadvantaged families.

## 1. Introduction

Family hardship is a multidimensional concept describing material conditions, social position, and economic position that are below the minimally acceptable level [1,2]. Family hardship is associated with a multitude of physical and psychosocial problems across age groups. For example, a previous study found disparities in early language trajectories within a low-income sample of children as a function of maternal education [3], suggesting that household income and parental education level can have multiplicative effects on child development. It is also known that looking after family members who live with chronic conditions can be stressful and increase family burden [4]. The overall family stress level would likely escalate when multiple members are suffering from chronic conditions [5]. Furthermore, the degree of hardship can be influenced by the role of affected members in the family. There is evidence showing that maternal mental and physical diseases had differential impacts on children’s health-related quality of life and behavior [6], and paternal job stress was associated with maternal frequent use of child physical punishment [7]. Thus, demographic characteristics such as disease history, income, and education level are widely used as indicators of family hardship. 

In terms of approaches to capture the heterogeneity in the family hardship profile, multidimensional approaches that analyze different indicators to determine the most appropriate set of group memberships have several advantages over unidimensional approaches. For example, one multidimensional approach is to first assign a weighting factor to each indicator based upon either expert opinions or through principal component analysis and then aggregate the indicators as a composite socioeconomic status (SES) index [8]. However, this approach cannot reveal the relative importance of different indicators to the overall socioeconomic profile. As research has demonstrated differences in the concepts of poverty and low income [9], and not all poor people would have applied for government financial assistance [10], the composite score approach is not ideal for examining such conceptual and profile differences. An alternative approach is to use latent class analysis (LCA), which is a subset of structural equation modeling for identifying latent variables (classes) based on the observed characteristics. It does not require an a priori classification decision rule or information on the relevance of variables for clustering individuals with similar profiles in homogenous groups [11]. The identified latent classes are mutually exclusive and exhaustive in that they have different item-response probabilities (i.e., the likelihood that the class members would fall within each specific indicator category). This method is particularly useful for analyzing individual responses to a particular exposure in connection with complex social concepts such as family hardship. 

Since December 2019, the coronavirus disease 2019 (COVID-19) has placed an unprecedented burden on communities and people across the world. A growing body of research has demonstrated the direct effect of transmission of Severe Acute Respiratory Syndrome Coronavirus 2 (SARS-CoV-2) on health and wellbeing [12]. The pandemic also has indirect influences on non-infected individuals and families, for example, through new practices and lifestyle changes as a result of infection control measures [13]. There have been debates as to why people react differently to these pandemic-related measures. Some evidence suggests that high-income individuals have a greater tendency to comply with these measures, whereas low-income individuals encounter more challenges to fight against the pandemic [14]. Compared to other population subgroups, children and parents may experience more challenges due to newly evolved stressors that are unique to the COVID-19 pandemic, such as unexpected school closure and online learning requirements. As shown in previous studies on the COVID-19 pandemic [15,16,17], elevated psychosocial problems among children and their parents are associated with multiple sociodemographic factors, including pre-pandemic family health conditions, job loss, migration background, limited living space, living with a single parent, and low family income. Although family hardship conditions tend to occur jointly rather than apart from each other, existing research has generally examined family hardship indicators as separate and independent family characteristics. There remains a limited understanding of what constitute a high-risk family hardship profile and the consequences of living with such a profile under the COVID-19 pandemic. Furthermore, due to social contact restrictions, many COVID-19-related studies have used online survey to collect data, which may lead to biased sampling such as the over-representation of better-off or tech-savvy families [18]. Research using mixed methods to collect survey data is thus needed to expand the current scope of literature on individual differences in pandemic responses.

Hong Kong has a high Gini coefficient (i.e., the degree of inequality in income/wealth across a population) of 0.539 in 2016 [19], densely populated areas [20], and marked income differences at both individual and family levels across the entire territory. Although the COVID-19 caseload since its outbreak remains relatively low in Hong Kong when compared with other world regions [21], the whole population has undergone drastic changes in all aspects of everyday life because of the stringent infection prevention and control policies. A previous study found that disadvantaged adults in Hong Kong had worse mental health during COVID-19 than their peers partly because of worry and job loss/instability [22], but evidence about disadvantaged children and their parents remains scarce. Hence, this study aimed to use the LCA techniques to identify subtypes of families living in the disadvantaged districts of Hong Kong based on patterns of family hardship. It also compared the patterns of parenting behavior, lifestyle practices, and wellbeing under the COVID-19 pandemic between the identified latent classes. 

## 2. Methods

This study surveyed families of preschoolers living in two districts (Sham Shui Po and Yuen Long) of Hong Kong during March to June 2020 (i.e., the second wave of COVID-19 in Hong Kong) when face-to-face classes were suspended [23]. We chose these two districts because of the uneven distribution of household income within these districts. In 2020, the median Hong Kong household income was HK$34,500, whereas the median household income in Sham Shui Po and Yuen Long districts was HK$30,000 and HK$30,800, respectively [20]. Although the median household income at the district level was fairly similar to the population level, the 2015 population statistics showed that nearly 20% of the recipients of comprehensive social security assistance (CSSA), which is a welfare program providing financial assistance to needy individuals and families in Hong Kong, were living in these two districts [24].

Specifically, we identified 32 under-resourced kindergartens from a total of 137 kindergartens in both districts using a grading checklist that evaluated (1) teacher qualification(s) and teaching experience; (2) teacher/student ratio; (3) school campus total area, classroom size, outdoor space, play areas; (4) teaching facilities, including the reading corner, books, toys, and number of available electronic devices; (5) school fee; (6) socio-economic background of students reflected by the percentage of families (i) receiving government social-security assistance, (ii) receiving a school fee remission, and (iii) having parents with a low education background; and (7) additional school support, including subsidies, financial, social service, and training. The overall percentage of preschool students receiving a school free remission (i.e., those students requiring governmental financial assistance in order to receive pre-primary education) in Hong Kong was 14% at the time of this study, whereas the percentage for our selected kindergartens was 53.1% (ranging from 22.7% to 72.6%). We subsequently invited principals and teachers of 32 kindergartens in these two districts to assist in subject recruitment, and they all accepted our invitation. We offered parents the option to complete the survey either online or through a paper and pen format at home. All the study measures were administered in Traditional Chinese. Upon obtaining school consent, the teachers were instructed to provide either the e-survey link or paper questionnaires to each student depending on their parent’s preference. The e-survey database automatically recorded each response in a safe and secure manner, whereas parent-completed paper questionnaires were collected by schools and subsequently sent to the research office for further processing. According to the information provided by the schools, 5705 parents were invited, and 1338 (20.0%) returned the completed questionnaires. The responding parents were usually the mother (98%), and all were competent at reading and comprehending our questionnaires. This study was approved by the ethics committee of the Institutional Review Board of the Hong Kong University/Hospital Authority Hong Kong West Cluster (Reference UW 20–177). Informed consent was obtained from the parents of all participating families.

## 3. Measures

### 3.1. Family Hardship

Based on evidence from the previous literature and their relevance to the local context [15], we included 11 binary variables indicating the degree of family hardship. These variables addressed the following socioeconomic and disease status: chronic disease (mother, father, and child), special education needs (child), mental disorder (mother and father), not yet completing secondary education (mother and father), parental marital status, monthly household income, and CSSA recipients. 

### 3.2. Child Maltreatment

Child maltreatment episodes in the preceding three months were reported by the parents using the Parent-Child Conflict Tactics Scale (CTS-PC) [25]. The CTS-PC, which has been used in previous local research [18] and demonstrated satisfactory reliability and validity for identification of child maltreatment victims in Hong Kong [19,20], consists of 13 items on physical assaults with subscales concerning corporal maltreatment, severe physical maltreatment, and very severe physical maltreatment; five items on psychological aggression; five items on neglect; and four items on non-violent discipline. 

### 3.3. Child Psychosocial Problems

Child psychosocial problems during school closure were measured using the parent version of the Strength and Difficulties Questionnaire (SDQ) for 2- to 4-year-olds [26], which has been widely used in Hong Kong local research [27]. The SDQ has 4 problem behavior scales assessing emotional symptoms (5 items), conduct problems (5 items), hyperactivity (5 items), and peer problems (5 items) on a 3-point scale from 0 = not true to 2 = certainly true. The internalizing problem score was computed by summing the scale scores of emotional symptoms and peer problems, whereas the scale scores of conduct problems and hyperactivity were summed to an externalizing problem score. 

### 3.4. Parenting Stress

Parenting stress during school closure was self-reported by the parent using the 17-item Parental Stress Scale (PSS) [28], which measures their subjective feelings of strains, difficulties, and dissatisfaction as a parent under the COVID-19 pandemic on a 6-point scale ranging from 1 = strongly disagree to 6 = strongly agree [7].

### 3.5. Child Physical Wellbeing 

Child physical wellbeing during school closure was reported by the parent using the physical functioning scale of the Pediatric Quality of Life Inventory™ 4.0 generic core scales (PedsQL™) [29], which has been extensively used in various Hong Kong Chinese pediatric populations [15]. The PedsQL™ physical functioning scale has 8 items measuring parents’ perceptions of their children’s physical functioning under this pandemic on a 5-point scale from 0 = never to 4 = almost always. Item ratings were linearly transformed into a 0–100 scale. We took the sum of all item ratings divided by the total number of scale items to calculate the total physical wellbeing score. 

### 3.6. Parent-Child Activities

The parent was asked to report the weekly frequency of recreational and learning activities with their children during school closure using the Chinese Parent-Child Interaction Scale (CPCIS), which was developed in Hong Kong and demonstrated good psychometric properties among local preschoolers [30]. The CPCIS has 8 items (arithmetic/mathematics, English alphabet, Chinese characters, reading, drawing, singing, storytelling, and discussing news and current affairs) for two subscales (recreational activities and learning activities) on a 4-point scale from 0 = never to 3 = 4 times or above each week. 

### 3.7. Child Lifestyle Practices 

Children’s lifestyle practices were measured in three aspects, namely exercise, sleep, and screen use (for learning or gaming purpose). The parent answered the question, “how long (in hours) did your child perform [the activity of interest] during a weekday/weekend?” The average amount of daily time spent on each activity was calculated by averaging the parent proxy-reported amount of time spent on the activity of interest during weekends and weekdays using the weighted average formula ([2 × weekend + 5 × weekday] ÷ 7).

### 3.8. Demographic Covariates

Child sex (male, female) and age were reported by the parent. We included these variables as covariates in the regression models.

## 4. Data Analysis

Descriptive statistics for family hardship indicators, parenting and lifestyle practices, demographic characteristics, and wellbeing scores were reported as mean and standard deviation for continuous variables and frequencies and percentages for dichotomous variables upon checking the distribution of data. Missing values on each variable of interest ranged from 0.7% to 60%. In order to minimize the bias attributable to missing information, missing data were imputed at the item level by means of multiple imputation using the MICE package in R [31]. All analysis variables were included in the imputation model, wherein each variable with missing data is dependent upon the value of all the other included variables. 

LCA was conducted using the R package “poLCA”, which estimates model parameters with maximum likelihood techniques using expectation-maximization and Newton–Raphson algorithms [32]. The optimal number of classes was determined based on three rules. First, the best fit model is selected based on the statistical fit indices [33], Akaike Information Criteria (AIC), Bayesian Information Criteria (BIC), and adjusted BIC. Lower values of BIC and AIC indicate better-fitting models [34]. Second, the classes should be distinct, meaningful, and theory-based [34]. The third rule is the parsimony interpretability of the latent class solutions. In other words, simple models with fewer classes are preferable. However, latent classes with less than 5% of the total sample are not considered due to the possibility of class over-extraction in the presence of non-normal data [35] and poor generalizability [36]. The maximum number of iterations (*maxiter*) through which the estimation algorithm cycled was 3000. To automate the search for the global—rather than the local—maximum of the log-likelihood function, the number of repetitions (*nrep*) was set to 30. Multinominal logistic regressions were conducted to generate an effect estimate, odd ratio (OR), for the association of membership in different latent classes with child maltreatment variables, whereas generalized linear regressions were used to generate an effect estimate, unstandardized regression coefficient (β), for the association of class membership with lifestyle practices and wellbeing scores. Analyses were adjusted for possible confounding effects of child age and gender. Statistical significance was set at a 2-sided *p* < 0.05. 

## 5. Results

### 5.1. Demographics

The total sample comprised 1338 preschoolers, with 790 (59%) completing the paper survey and 548 (41%) completing the e-survey (Table 1). The mean age was 4.83 years (SD = 0.99). There were slightly more girls (girls: 53.1% vs. boys: 46.9%). In total, 14.4% had special educational needs and 7.6% had chronic diseases. Regarding their parental disease history, 2.4% had mothers with chronic diseases and 3.9% had mothers with mental disorders. On the other hand, 5.0% had fathers with chronic diseases and 0.9% had fathers with mental disorders. Most fathers and mothers had attained upper secondary education or above, and the majority were either cohabiting or married. The participating families had a relatively equal distribution in low-income classification (46.9% HK$19,999 or below), with 6.4% receiving government welfare assistance at the time of the survey. In total, 68.2% of the preschoolers had experienced corporal punishment, with the prevalence of physical maltreatment and severe physical maltreatment reported as 9.3% and 2.6%, respectively. Furthermore, 79.5% had experienced psychological aggression behavior, and 24% had episodes of neglect. 

### 5.2. Latent Class Analysis

**Figure 1:**
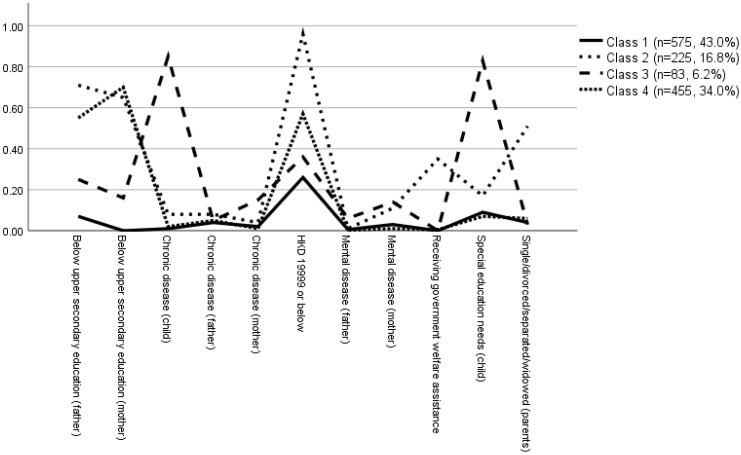
Item-response probabilities for 11 family hardship indicators across the 4 classes (note: Class 1 = Low hardship; Class 2 = Poverty; Class 3 = Poor health; Class 4 = Low income).

Five latent class models were estimated, and their fit indices are presented in Table 2. Overall, the four-class solution provided the best, most parsimonious fit based on statistical indices (as indicated by the AIC and BIC) and was also most meaningful conceptually. The four classes consisted of participants facing limited family hardship (class 1; low hardship characterized by the low likelihood of belonging to any of the family hardship indictor categories), participants living in poverty (class 2; poverty characterized by the high likelihood of having low family income together with government welfare assistance), participants with high disease burden at the family level (class 3; poor health characterized by the high likelihood of family members having medical diagnoses), and participants living in low-income households (class 4; low income characterized by the relatively high likelihood of having a low family income yet without government welfare assistance). The prevalence rates of the four latent classes are reported in Table 3 and Figure 1. Consistent with a previous publication [37], a high probability of item endorsement was defined as a value greater than 0.50.

### 5.3. Comparison of the Four Classes on Parenting Behavior, Lifestyle Practices, and Wellbeing under the COVID-19 Pandemic

Results of regression analyses (Table 4) indicated that compared to Class 1 children, Class 2 children were more likely to have neglect experiences (OR = 1.73, *p* = 0.013), faced higher parenting stress (β = 3.66, *p* = 0.001), displayed more externalizing (β = 0.52, *p* = 0.045) and internalizing problems (β = 0.59, *p* = 0.008), and enjoyed fewer parent-child learning (β = −0.14, *p* = 0.012) and recreational activities (β = −0.18, *p* = 0.001). On the other hand, compared to Class 1 children, Class 3 children were more likely to have psychological regression experiences (OR = 1.99, *p* = 0.035), showed more externalizing problems (β = 0.73, *p* = 0.048), and spent less time exercising (β = −0.57, *p* < 0.001). Lastly, compared to Class 1 children, Class 4 children were less likely to have psychological aggression experiences (OR = 0.76, *p* = 0.048), faced higher parenting stress (β = 1.90, *p* = 0.020), had better physical wellbeing (β = 2.11, *p* = 0.011) and longer daily sleep duration (β = 0.13, *p* = 0.017), and enjoyed fewer recreational activities (OR = −0.16, *p* < 0.001).

## 6. Discussion

This study demonstrated the feasibility of using sociodemographic and disease characteristics of parents and children as a basis for the classification of family hardship subtypes. We identified four distinct and interpretable subtypes of family hardship based on 11 socioeconomic and disease indicators. The identified family classes were labeled as low hardship, poverty, poor health, and low income, respectively. The results highlight the severity of wealth and income disparities among socially disadvantaged families in Hong Kong. In addition, there were notable differences in parenting behavior, lifestyle practices, and wellbeing across these family classes. This investigation extends previous findings by showing that the poorest of the poor are most vulnerable to the effects of disease outbreaks and disasters [38]. 

Previous studies have shown that family poverty and parental depressive symptoms were associated with child neglect and psychological abuse [16,39]. Echoing previous research, this study revealed that children in the *low hardship* class had lower risks of being neglected than those in the *poverty* class, and lower risks of being psychologically aggressed than those in the *poor health* class. However, when compared to those in the *low income* class, children in the *low hardship* class had higher odds of being psychologically aggressed, even after adjusting for child age and gender. A possible explanation is that when compared to the *low income* class, parents in the *low hardship* class may spend more time with children at home during the pandemic, thereby increasing the likelihood of parent-child conflicts. In this study, although all children were living in disadvantaged neighborhoods, the *low hardship* and *low income* classes differed in the aspects of family income and parental education level. A previous study has posited that the pandemic not only exacerbates pre-existing social inequalities but also creates new forms of disparities [40]. Building upon this notion, our results suggest that the pandemic can aggravate the impact of even subtle pre-existing socioeconomic differences on children and parents. Instead of certain risk factors, the overall family profile can thus better indicate the degree of individual-level vulnerability to the COVID-19 impact. For example, members in both the *poverty* and *low income* classes have a relatively high probability of having low family income, but the *poverty* class, by comparison, has a more disadvantaged profile characterized by high probabilities of having government welfare assistance, single parent, and less educated/sick father. It is possible that such a profile, rather than low income per se, accounts for the higher risk of child neglect in the *poverty* class than the *low hardship* class. 

Consistent with previous findings about the association between poverty and poor health [41,42], this study found that families in the *poverty* class experienced higher levels of children’s externalizing and internalizing problems and greater parenting stress compared to those in the *low hardship* class, suggesting that adjustment difficulties could be among the factors associated with the increased occurrence of child neglect in poor families under this pandemic. On the other hand, parents in both *poor health* and *low hardship* classes had similar stress levels, but children in the *poor health* class exhibited significantly higher levels of externalizing problems, suggesting that children’s behavioral issues could also be a possible reason for the occurrence of psychological aggression in families with a high disease burden under the COVID-19 pandemic. As shown in previous research [43], parents who have a higher risk of engaging in parent-child aggression tend to give harsh responses to noncompliant child behavior. Future research should examine whether parents of less healthy children have poorer emotional regulation skills, which, in turn, increase their risk of engaging in child emotional abuse during disease outbreaks. 

Interestingly, although parenting stress was higher in the *low income* class than the *low hardship* class, children in the *low hardship* class were more likely than those in the *low income* class to suffer psychological aggression. This could be because our data captured parenting stress but no other types of stress. For example, the better-off families may experience decreases in income or net profit during the pandemic [44], which, in turn, can intensify their financial stress and risk of child emotional abuse during the same period. Furthermore, children in the *low hardship* class were found to have poorer physical well-being than those in the *low income* class, suggesting that children in better-off families are also vulnerable to the physical health impact of the pandemic.

In addition, we explored the activity patterns and lifestyle practices of children in different family hardship classes. The findings of lower levels of parent-child recreational activities in both the *poverty* and *low income* classes and lower levels of parent-child learning activities in the *poverty* class relative to the *low hardship* class are consistent with the current literature regarding social deprivation that could result from physical distancing measures [45] and provide evidence for family income and poverty as powerful correlates of family interactions and wellbeing during COVID-19. By contrast, the finding of longer daily sleep duration among children in the *low income* class relative to those in the *low hardship* class suggests that children in better-off families could have a higher risk of inadequate sleep during school closure. We also observed shorter daily exercise duration among children in the *poor health* class compared to those in the *low hardship* class, indicating that families with a high disease burden need more guidance or motivational resources to engage young children in physical activities during the pandemic. Previous research has reported that people with mental health problems or disabilities are more affected by the pandemic [46]. Public resources should be prioritized to help these vulnerable individuals to overcome the challenges and difficulties brought by the pandemic.

This study has several limitations that should be considered when interpreting the results. First, all data were based on parent self-report or proxy-report questionnaires, and some indictors had a relatively large proportion of missing values. Although multiple imputation was used to fill in the missing values, the uncertainty of parameter estimation cannot be ruled out. Future research should ascertain the associations observed in this study using multiple data sources, such as multiple informant reports or electronic health records, to enhance data availability and reliability. Second, it is important to note that although families were recruited from disadvantaged neighborhoods, they were not representative of the whole Hong Kong population. Therefore, caution is needed when generalizing our results to other populations. Third, due to the cross-sectional design, we have no pre-pandemic data on the variables of interest, and thus we cannot conclude whether the observed variations in parenting and lifestyle practices and wellbeing between family hardship classes emerged following the pandemic. Fourth, our LCA model focused on socioeconomic and disease characteristics. Other factors, such as academic and job pressures, social support, and access to public services and facilities, could be explored in future studies. 

## 7. Conclusions

The present study identified four classes of family hardship and highlighted their associations with parenting behavior, lifestyle practices, and wellbeing during the COVID-19 pandemic. Class 2 (*poverty*) showed higher odds of child neglect, demonstrated more parental and child adjustment problems, and had fewer parent-child activities than Class 1 (*low hardship*). Class 3 (*poor health*) demonstrated similar patterns except lifestyle practices with children having a shorter daily exercise duration relative to Class 1. Class 4 (*low family income*) had lower odds of psychological aggression towards children but higher parenting stress and fewer parent-child recreational activities, although their children had higher levels of physical wellbeing and slept more than those in Class 1. The findings suggest a need to adopt different intervention strategies to mitigate the COVID-19-related health inequalities among those in poverty and other families in general. 

## Figures and Tables

**Table 1:** Description of the study sample (*n* = 1338) and comparison of observed and imputed data.

Variables	Observed	Imputed
Age (in years), mean (SD)	4.83 (0.99)	4.76 (1.02)
Gender, *n* (%)		
Boy	617 (46.9)	721 (53.9)
Girl	699 (53.1)	617 (46.1)
Mother having chronic disease, *n* (%)		
Yes	32 (2.4)	35 (2.6)
No	1285 (97.6)	1303 (97.4)
Father having chronic disease, *n* (%)		
Yes	66 (5.0)	66 (4.9)
No	1249 (95.0)	1272 (95.1)
Child having chronic disease, *n* (%)		
Yes	101 (7.6)	101 (7.5)
No	1227 (92.4)	1237 (92.5)
Child having special educational needs, *n* (%)		
Yes	190 (14.4)	190 (14.2)
No	1127 (85.6)	1148 (85.8)
Mother having mental disorder, *n* (%)		
Yes	52 (3.9)	57 (4.3)
No	1268 (96.1)	1281 (95.7)
Father having mental disorder, *n* (%)		
Yes	12 (0.9)	12 (0.9)
No	1306 (99.1)	1326 (99.1)
Maternal education level, *n* (%)		
Below upper secondary education	434 (33.5)	475 (35.5)
Upper secondary education or above	863 (66.5)	863 (64.5)
Paternal education level, *n* (%)		
Below upper secondary education	416 (32.5)	471 (35.2)
Upper secondary education or above	864 (67.5)	867 (64.8)
Parental marital status, *n* (%)		
Cohabited/married	1169 (91.5)	1169 (87.4)
Single/divorced/separated/widowed	108 (8.5)	169 (12.6)
Monthly household income, *n* (%)		
HKD 19,999 or below	597 (46.9)	652 (48.7)
Above HKD 19,999	676 (53.1)	686 (51.3)
Receiving government welfare assistance, *n* (%)		
Yes	80 (6.4)	80 (6.0)
No	1177 (93.6)	1258 (94.0)
Severe physical maltreatment, *n* (%)		
Yes	32 (2.6)	131 (9.8)
No	1207 (97.4)	1207 (90.2)
Physical maltreatment, *n* (%)		
Yes	115 (9.3)	218 (16.3)
No	1118 (90.7)	1120 (83.7)
Corporal punishment, *n* (%)		
Yes	836 (68.2)	891 (66.6)
No	389 (31.8)	447 (33.4)
Non-violent discipline, *n* (%)		
Yes	1090 (89.2)	1090 (81.5)
No	132 (10.8)	248 (18.5)
Psychological aggression, *n* (%)		
Yes	975 (79.5)	975 (72.9)
No	252 (20.5)	363 (27.1)
Neglect, *n* (%)		
Yes	297 (24.0)	392 (29.3)
No	941 (76.0)	946 (70.7)
Externalizing problems (0–20), mean (SD)	6.99 (3.19)	7.03 (3.10)
Internalizing problems (0–20), mean (SD)	4.90 (2.76)	4.96 (2.69)
Parenting stress (17–102), mean (SD)	48.03 (11.24)	49.82 (13.29)
Physical wellbeing (0–100), mean (SD)	87.34 (13.09)	87.18 (13.39)
Parent-child learning activities (0–3), mean (SD)	1.97 (0.67)	1.97 (0.69)
Parent-child recreational activities (0–3), mean (SD)	1.63 (0.63)	1.58 (0.67)
Sleep duration (hour/day), mean (SD)	10.84 (0.87)	11.08 (0.89)
Exercise duration (hour/day), mean (SD)	1.21 (0.69)	1.83 (0.96)
Electronic device use for learning (hour/day), mean (SD)	1.09 (0.83)	2.25 (1.07)
Electronic device use for gaming (hour/day), mean (SD)	1.14 (1.06)	2.27 (1.13)

**Table 2:** Summary of latent class model identification and fit statistics.

No. of Classes	AIC	BIC	Adjusted BIC	Smallest Class, %	Entropy
1	10,241.3	10,298.5	10,263.5	-	-
2	9706.1	9825.7	9752.6	41.7%	0.613
3	9535.7	9717.6	9606.4	5.5%	0.654
**4**	**9465.0**	**9709.3**	**9560.0**	**5.9%**	**0.633**
5	9430.8	9737.6	9550.1	3.7%	0.690

*Note.* AIC, Akaike Information Criterion; BIC, Bayesian Information Criterion. Bolded row represents the identified model.

**Table 3:** Four-class model: estimated probabilities by latent class membership ^a^.

Family Hardship Indicators	Class 1	Class 2	Class 3	Class 4
	Low hardship	Poverty	Poor health	Low income
	(*n* = 611, 45.7%)	(*n* = 187, 14.0%)	(*n* = 79, 5.9%)	(*n* = 461, 34.5%)
Having chronic disease (mother)	0.016	0.044	**0.151**	***0.008***
Having chronic disease (father)	***0.039***	**0.079**	0.053	0.048
Having chronic disease (child)	***0.008***	0.083	**0.846**	0.018
Having special education needs (child)	0.086	0.172	**0.833**	***0.073***
Having mental disorder (father)	0.006	0.013	**0.056**	***0.003***
Having mental disorder (mother)	0.027	0.115	**0.136**	***0.010***
Below upper secondary education (mother)	***0.000***	0.649	0.157	**0.695**
Below upper secondary education (father)	***0.075***	**0.709**	0.246	0.546
Parental marital status: single/divorced/separated/widowed	0.042	**0.509**	***0.031***	0.062
Monthly household income: HKD 19,999 or below	***0.256***	**0.959**	0.356	0.572
Receiving government welfare assistance	***0.000***	**0.347**	0.000	0.005

^a^ Bolded indices are the highest probabilities (underlined) and the lowest probabilities (italicized) in the rows.

**Table 4:** Regression analyses indicating the relationship between family hardship classes and parenting and lifestyle practices and wellbeing under the COVID-19 pandemic.

	Class 2: Poverty (vs. Low Hardship)		Class 3: Poor Health (vs. Low Hardship)		Class 4: Low Income (vs. Low Hardship)	
	OR (95%CI)	*p*-Value	OR (95%CI)	*p*-Value	OR (95%CI)	*p*-Value
Severe physical maltreatment ^a^	1.49 (0.91, 2.45)	0.113	0.73 (0.30, 1.75)	0.474	0.86 (0.56, 1.32)	0.480
Physical maltreatment ^a^	1.19 (0.77, 1.83)	0.438	1.32 (0.73, 2.38)	0.359	0.97 (0.69, 1.35)	0.837
Corporal punishment ^a^	1.15 (0.81, 1.64)	0.421	1.26 (0.75, 2.09)	0.382	1.27 (0.98, 1.64)	0.074
Non-violent discipline ^a^	0.79 (0.52, 1.20)	0.268	1.26 (0.64, 2.47)	0.499	0.76 (0.56, 1.04)	0.083
Psychological aggression ^a^	1.09 (0.75, 1.59)	0.661	1.99 (1.05, 3.78)	0.035	0.76 (0.58, 0.998)	0.048
Neglect ^a^	1.73 (1.13, 2.67)	0.013	1.25 (0.70, 2.25)	0.454	0.77 (0.52, 1.15)	0.206
	β (95%CI)	*p*-value	β (95%CI)	*p*-value	β (95%CI)	*p*-value
Externalizing problems	0.52 (0.01, 1.02)	0.045	0.73 (0.01, 1.45)	0.048	−0.08 (−0.45, 0.30)	0.694
Internalizing problems	0.59 (0.15, 1.03)	0.008	0.54 (−0.09, 1.16)	0.094	−0.14 (−0.47, 0.18)	0.391
Parenting stress	3.66 (1.50, 5.83)	0.001	2.07 (−1.02, 5.17)	0.189	1.90 (0.30, 3.50)	0.020
Physical wellbeing	−0.08 (−2.26, 2.10)	0.945	−1.20 (−4.32, 1.91)	0.450	2.11 (0.49, 3.72)	0.011
Parent-child learning activities	−0.14 (−0.26, −0.03)	0.012	−0.07 (−0.23, 0.09)	0.388	−0.08 (−0.17, 0.00)	0.050
Parent-child recreational activities	−0.18 (−0.29, −0.07)	0.001	−0.06 (−0.21, 0.10)	0.461	−0.16 (−0.24, −0.08)	<0.001
Sleep duration (hour/day)	0.12 (−0.02, 0.27)	0.102	0.19 (−0.01, 0.40)	0.066	0.13 (0.02, 0.24)	0.017
Exercise duration (hour/day)	0.07 (−0.09, 0.23)	0.378	−0.57 (−0.79, −0.35)	<0.001	0.04 (−0.08, 0.15)	0.515
Electronic device use for learning (hour/day)	−0.002 (−0.18, 0.17)	0.979	−0.004 (−0.25, 0.25)	0.974	−0.02 (−0.15, 0.11)	0.713
Electronic device use for gaming (hour/day)	0.10 (−0.08, 0.29)	0.267	−0.04 (−0.30, 0.23)	0.796	0.12 (−0.02, 0.26)	0.085

^a^ Reference group: children without the exposure of interest; Note: All regression analyses were controlled for child age and gender.

## Data Availability

The data that support the findings of this study are available on request from the corresponding author.
